# *Anoxybacillus suryakundensis* sp. nov, a Moderately Thermophilic, Alkalitolerant Bacterium Isolated from Hot Spring at Jharkhand, India

**DOI:** 10.1371/journal.pone.0085493

**Published:** 2013-12-20

**Authors:** Kamal Deep, Abhijit Poddar, Subrata K. Das

**Affiliations:** Institute of Life Sciences, Department of Biotechnology, Bhubaneswar, India; Belgian Nuclear Research Centre SCK/CEN, Belgium

## Abstract

Four closely related facultative anaerobe, moderately thermophilic, Gram positive rods (JS1^T^, JS5, JS11, and JS15) were isolated from sediment samples from a hot spring at Suryakund, Jharkhand, India. Colonies were pale yellow, rough surface with uneven edges on TSA after 72 h incubation. Heterotrophic growth was observed at 40-60°C and pH 5.5-11.5; optimum growth occurred at 55°C and pH 7.5. 16S rRNA gene sequence analysis revealed the strains belong to genus *Anoxybacillus*. DNA-DNA homology values among strains were above 70% and showed distinct ERIC and REP PCR profile. On the basis of morphology and biochemical characteristics, strain JS1^T^ was studied further. Strain JS1^T^ showed 99.30% sequence similarity with *A. flavithermus* subsp. *yunnanensis*, 99.23% with *A. mongoliensis*, 99.16% with *A. eryuanensis*, 98.74% with *A. flavithermus* subsp. *flavithermus*, 98.54% with *A. tengchongensis*, 98.51% with *A. pushchinoensis*, 97.91% with *A. thermarum*, 97.82% with *A. kaynarcensis*, 97.77% with *A. ayderensis* and *A. kamchatkensis*, 97.63% with *A. salavatliensis*, 97.55% with *A. kestanbolensis*, 97.48% with *A. contaminans*, 97.27% with *A. gonensis* and 97.17% with *A. voinovskiensis*. In 16S rRNA secondary structure based phylogenetic comparison, strain JS1^T^ was clustered with *Anoxybacillus eryuanensis*, *A. mongoliensis*, and *A. flavithermus* subsp. *yunnanensis* and showed 15 species specific base substitutions with maximum variability in helix 6. Moreover, DNA-DNA relatedness between JS1^T^ and the closely related type strains were well below 70%. The DNA G+C content was 42.1 mol%. The major fatty acids were C_15:0 iso_, C_16:0 iso_ and C_17:0iso_. The polar lipids were a phosphatidylgylycerol, a diphosphatidylglycerol, a phosphatidylethnolamine, a phosphatidylcholine, a phosphatidyl monomethylethanolamine and four unknown lipids. Based on polyphasic approach, strain JS1^T^ represent a novel species of the genus *Anoxybacillus* for which *Anoxybacillus suryakundensis* sp. nov. is proposed. The type strain is JS1^T^ (= DSM 27374^T^ = LMG 27616^T^ =JCM19211^T^).

## Introduction

The genus *Anoxybacillus* was introduced by Pikuta et al. that comprised members showing anaerobic growth [1]. Later it was found that *Anoxybacillus pushchinoensis* strain K1^T^ representing the genus *Anoxybacillus* can grow aerobically in ANX medium at pH 9-9.7 that led to amendment of genus description [2]. Members are Gram-positive, spore forming rods of size varying 0.4-0.85 x 2.5-5.0 µm, aerotolerant anaerobes or facultative anaerobes. Oxidase and catalase reactions are variable. DNA G+C content varies between 42-57 mol%. At the time of writing this manuscript, total 20 species was reported from different parts of the globe [1,3-14] being thermophilic and alkalitolerant. 

Exploration of new microbes in hot springs is of importance owing to the intriguing biogeochemistry and extreme conditions of these environments. Extreme habitat inspired several studies on hot spring microbial diversity throughout the world [15,16]. Microorganisms that survive in hot springs have unique adaptations to high temperature and are an important bio-resource. Our recent studies revealed the presence of new species of bacterium from different tropical hot springs in India [17-20]. These observations inspired us to explore the microbial diversity in other hot springs. 

In the present study we describe the phenotypic and genotypic characteristics of a novel strain, designated as JS1^T^, isolated from a hot spring at Suryakund, Jharkhand, India. Based on this polyphasic evidence, it is proposed that JS1^T^ be assigned as the type strain of the novel species *Anoxybacillus suryakundensis* sp. nov.

## Materials and Methods

### Sample collection and isolation of bacterium

Sediment samples were collected twice from a hot spring at Suryakund ((24°08′58″N, 85°38′44″E), Jharkhand, India. The surface temperature of the sediment sample at the site of collection was 80°C and the pH was 7.4. No specific permissions were required for the collection of samples from this hot spring. Moreover, this location did not involve endangered and protected species and it is not under regulatory body concerned with protection of wildlife. Samples collected in a sterile container were transported to the laboratory without temperature control. 2.0 g (wet weight) sediment sample was transferred into a 250 ml conical flask containing 50 ml trypticase soy broth (Difco) pH 7.2 and incubated on a shaker (ISF-I-V; Adolf Kuhner AG) at 55°C and 200 rpm After overnight incubation, the suspension was serially diluted and plated onto trypticase soy agar (Difco) plates pH 7.2 and incubated at 55°C for three days. Morphologically similar pale yellow, rough surface circular colony that appeared on trypticase soy agar plate were picked and purified by repeated streaking on the same medium. For short term preservation, isolates were streaked on trypticase soy agar and stored at 4°C. For long-term preservation, the culture was stored at -80°C in 15% (v/v) glycerol. 

For comparative study, closely related type strains were procured from Leibniz-Institut DSMZ-Deutsche Sammlung von Mikroorganismen und Zellkulturen GmbH, Germany, National Collection of Industrial, Marine and Food Bacteria (NCIMB), UK, Korean Collection for Type Cultures (KCTC), Korea and from BCCM/LMG Bacteria collection, Universiteit Gent, Belgium. Unless indicated otherwise, all strains were grown and maintained as mentioned previously.

### Phenotypic, biochemical and physiological characterization

Cell morphology was examined by transmission electron microscopy (model FEI Morgagni 268D) as described by Jyoti et al. [18]. Gram character was determined using Gram staining kit (Becton-Dickinson, USA) following manufacturer’s instruction. The formation of spores was observed by growing culture for 48 h in half strength TSB supplemented with 5 mg/l MnSO_4_, 4 H_2_O. Spores were detected by using Schaeffer and Fulton Spore Stain Kit (Hi-Media) following manufacturer’s instruction. For phenotypic characterization, all isolates and reference type strains were tested under same laboratory conditions. Tests like oxidase, catalase indole, methyl red, Voges Proskauer, lysine decarboxylase, ornithine decarboxylase, citrate and malonate utilization, hydrolysis of starch, urea, ONPG and nitrate reduction was performed by previously described methods [20]. Utilization of different carbohydrates as sole source of carbon and energy with concomitant production of acid was tested in peptone water basal media (Hi-Media) supplemented with substrates at concentration of 5.0 g/l and phenol red of 0.18 g/l. Tests were considered positive, weakly positive and negative based on the media colour (yellow, light yellow or pink and red respectively). In addition to classical tests, oxidation of organic compounds as sole source of carbon was determined using API50CH, API20E (bioMérieux) and Hi25 identification kit (Hi-Media) following manufacturer’s instruction except incubation was performed at 55°C for 18 h. Yeast extract-peptone broth containing 0-5.0% (w/v) NaCl was inoculated and incubated at 55°C for 4 days to test for salt tolerance. Ethanol tolerance was determined by growing the cells in TSB medium containing ethanol at a final concentration of 0-15% (v/v). Anaerobic growth was tested using the BD GasPak EZ system (Becton-Dickinson). The temperature for growth in the range of 30-60°C was examined in TSB medium at pH 7.5 for 2 days. Similarly, the pH range for growth was examined in TSB medium prepared in buffered solutions [21] in the pH range 3.5-11.5 in steps of 0.5 pH unit at 55°C for 48 h.

### DNA isolation, PCR amplification and sequencing of 16S rRNA

Isolation of chromosomal DNA, amplification and sequencing of 16S rRNA gene of the isolates were performed by the methods described earlier [22]. Primers used for the amplification of 16S rRNA were 5’-GAG TTT GAT CCT GGC TCA-3’ (forward primer) and 5’-AGA AAG GAG GTG ATC CAG CC-3’ (reverse primer) [23]. The nucleotide sequences thus obtained were assembled using the sequence alignment editor program Bioedit (http://www.mbio.ncsu.edu/Bio-Edit/bioedit.html) and was compared with those in GenBank after BLAST searches [24] and using the EzTaxon-e server [25]. 16S rRNA sequences of valid strains obtained from EzTaxon-e server were considered for secondary structure based phylogenetic analysis where *E.coli* 16S rRNA was used as reference template. All sequences were aligned using template based alignment algorithm CRWalign [26], edited and refined manually. The base positions were assigned according to *E.coli* numbering and helix numbering was adopted from ARB database [27]. The 16S rRNA based phylogenetic tree was constructed according to the Kimura two-parameter model using the MEGA 5 [28] software package (The Biodesign Institute, Arizona, USA). A phylogenetic tree was constructed using the neighbor joining method of Saitou & Nei [29] and with the maximum likelihood algorithms. The statistical significance of branch points was calculated by 1,000 bootstrap re-samplings of the data [30]. 

### Determination of DNA base ratio and DNA-DNA homology study

For determination of DNA G+C content, DNA was degraded enzymatically into nucleosides as described by Mesbah et al. [31]. The obtained nucleoside mixture was then separated by HPLC (Shimadzu) using an analytical column (Vydac 201 SP54, C18, 5μm; 250 x 4.6 mm) equipped with a guard column (201 GD54H; Vydac). Non methylated lambda phage DNA (Sigma) was used as the calibration reference.

DNA-DNA re-association study is considered as standard method for bacterial species delineation [32]. Hence, DNA-DNA re-association study was performed among the isolates and between strain JS1^T^ and other closely related type species showing 16S rRNA sequence similarity more than 97% following the method described by Ezaki et al. [33]. DNA from *E. coli* strain HB101 was taken as an unrelated negative control. Hybridization was performed following the methods described earlier [20].

### ERIC and REP-PCR

ERIC-PCR (enterobacterial repetitive intergenic consensus sequence PCR) and REP-PCR (repetitive extragenic palindromic PCR) was performed with the isolated strains by method as described previously [34]. For ERIC PCR, two oligonucleotide primers were 5ʹ-ATG TAA GCT CCT GGG GAT TCA C-3ʹ and 5ʹ-AAG TAA GTG ACT GGG GTG AGC G-3ʹ. For Rep PCR two (18-mer) oligonucleotide primers were REP 1R-1 (5ʹ-III ICG ICG ICA TCI GGC-3’) and REP-21 (5ʹ-ICG ICT TAT CIG GCC TAC-3ʹ) [35]. The fingerprints were analyzed by using Bionumerics software (Applied Maths, Bio-Rad). 

### Chemotaxonomic analysis

For the analysis of polar lipids, and nature of diaminoacids in the peptidoglycan layer, cells were grown in TSB medium to mid log phase in a rotary shaker at 55°C. Polar lipids were determined by 2D-TLC following the method of Bligh & Dyer [36]. Cellular fatty acids were extracted from stationary phase cells grown on TSA at 55°C for 3 days and analyzed by previously described method [19]. Diaminopimelic acid (DAP) and cell wall sugar was detected following the method of Staneck & Roberts [37] by using the TLC on cellulose plates (Merck, cat no. 1.05577.0001, Germany).

### Nucleotide sequence accession number

The GenBank/EMBL/DDBJ accession numbers for the 16S rRNA gene of strain JS1^T^, JS5, JS11 and JS15 are KC958552, KF772607, KF772610 and KF772608 respectively. 

## Results and Discussion

Abundance of bacterial population in the hot spring sample was very low for which enrichment procedure [5,12] was followed. Four pale yellow, rough surface circular colonies with uneven edges were picked for 16S rRNA sequence analysis in order to determine their phylogenetic position. 16S rRNA sequence of JS1^T^ (1435 nt), JS5 (1427 nt), JS11 (1396 nt), and JS15 (1458 nt) were obtained in this study. The levels of 16S rRNA gene sequence similarity among the isolates were between 97.13 and 99.43% (Table S1). Biochemical tests with all isolates exhibited similar phenotype except hydrolysis of starch and utilization of sugars (Table S2). DNA-DNA homology values were well above 70% (Table S1). Looking into the phenotypic characteristics, 16S rRNA sequence similarity and DNA-DNA homology values, all isolates can be considered to represent same species of the genus *Anoxybaciilus*. Although, heterogeneity among the isolates were confirmed by ERIC and REP-PCR that produced distinct banding pattern (Figure S1A & B) indicating all four isolates were of non clonal origin and strain JS1^T^ was considered for further characterization.

Strain JS1^T^ was non motile, straight rod of length 1-4 µm and diameter 0.3-0.8 µm (Figure S2). It formed spherical spores located centrally or terminally (Figure S3). Phenotypically all strains were negative for indole, Voges Proskauer, H_2_S production, arginine dihydrolase, ornithine decarboxylase, lysine decarboxylase, phenylalanine deaminase and urease activity. All strains were positive for utilization of fructose, sucrose, trehalose; negative for utilization of citrate and malonate. However, considering the phenotypic and chemotaxonomic properties, the strain JS1^T^ can be differentiated from other type strains of the genus *Anoxybacillus*. Phenotypic properties that differentiate strain JS1^T^ from other closely related strains are listed in Table 1. 

**Table 1:** Features that differentiate strain JS1^*T*^ with its closely related species of *Anoxybacillus*.

Features	1	2	3	4	5	6	7*^e^	8	9	10	11	12	13	14	15	16
Motility	-	+	+	+	+	+	-	+	+	+	+	+	+	+	-	+
Endospore																
Shape	S	E*^a^	O*^b^	E*^j^	E/Cy*^d^	E/Cy*^d^	S	E/C*^f^	S*^g^	O*^h^	E*^i^	S*^g^	E*^j^	S*^k^	ND	E*^m^
Position	T/C	T*^a^	T*^b^	T*^j^	T*^d^	T*^d^	T	T*^f^	T*^g^	T*^h^	T*^i^	T*^g^	T/ST*^j^	T*^k^	ND	C/ST*^m^
Swelling of sporangia	Slightly	ND	Slightly	+*^j^	ND	ND	-	ND	ND	ND	-	ND	Slightly*^j^	ND	ND	+*^m^
Biochemical tests																
Catalase	+	+	+	+	+	+	-	+	+	-	+	+	+	+	+	-
Oxidase	+	+	+	+	+	+	ND	-	+	-	+	+	-	+	+	-
Nitrate reduction	**-**	**-**	**-**	+	**-**	+	+	**-**	+	**-**	+	+	+	**-**	+	+
Methyl red	+	-	+	-	+	+	ND	-	-	-	-	-	+	-	-	+
Growth characteristics																
Temperature range (°C)	40-60	30-66	30-75	30-72	35-70	30-70	37-65	55–67	30-70	37-66	37-69	40-70	37- 60	40-70	30-64	35-70
Temperature optima (°C)	55	60	60	60-65	55	50	62	65	50	57-62	60	50-55	50	55-60	54	60
pH range	5.5-11.5	5.5-10*^a^	5.0-10.8*^b^	5.5-9*^c^	7-11*^d^	7-11*^d^	8-10.5	6-7.5*^f^	6-11*^g^	5.7-9.9*^h^	5.5-9.5*^i^	6-10.5*^g^	4-9.5*^j^	6-10*^k^	7-8*^l^	6-10*^m^
pH optima	7.5	7-7.5*^a^	8*^b^	7*^c^	8*^d^	8.5*^d^	9.5-9.7	7.2*^f^	7.5-8.5*^g^	6.8-8.5*^h^	8-9*^i^	7.5-8.5*^g^	7*^j^	7.5-8*^k^	ND	7*^m^
NaCl tolerance (%)	3.5	3.5	5	2.5	3	4	3	2.5	2.5	3	4.5	4	5	4	3	4
Ethanol tolerance (%)	2	12	2	2	2	4	ND	2	2	-	2	4	-	2	-	3
Hydrolysis of																
Gelatin	+	-	+	-	+	+	-	-	+	-	+	-	+	+	-	+
Starch	+	-	+	+	+	+	+	-	+	+	+	+	+	+	-	-
Esculin	+	+	-	+	+	+	ND	+	+	+	+	+	-	-	+	+
DNA	+	+	-	-	-	-	ND	-	+	-	+	+	-	+	+	+
Acid production from D-glucose	+	-	+	+	+	+	ND	-	+	+	+	+	+	+	+	+
Utilization of																
Glucose	+	+	+	+	+	+	+	-	+	+	+	+	+	+	+	+
Raffinose	-	w	w	-	-	-	-	-	-	-	-	+	+	+	+	
Mannose	+	w	w	+	+	+	-	+	+	+	+	+	+	w	+	
Mannitol	+	+	+	+	+	+	-	-	+	+	+	+	+	+	+	+
Lactose	+	-	-	-	-	-	-	-	-		-	-	-	-	-	
Galactose	-	+	+	+	-	+	-	-	+	+	+	+	+	+	+	
Xylose	-	+	+	-	-	-	-	-	+	-	-	-	+	+	+	+
Rhamnose	-	-	-	-	-	w	-	w	-	w	+	-	-	-	-	+
Maltose	+	+	+	+	+	+	ND	+	+	-	+	+	+	+	+	
Arabinose	-	+	-	w	-	-	ND	-	+	w	-	-	+	w	+	
G+C content (mol%)	42.1	42.3*^a^	44.2*^b^	41.6*^c^	42.6*^d^	41.4*^d^	42.2+/- 0.2	53.5*^f^	54*^g^	42.3*^h^	45.1*^i^	50*^g^	44.4*^j^	57*^k^	43.9*^l^	42.9*^m^

All data are from present study unless indicated.

Strains: 1, *A. suryakundensis* strain JS1**^T^**; 2, *A. flavithermus* subsp. *yunnanensis* DSM 23293**^T^**; 3, *A. mongoliensis* DSM 19169**^T^**; 4, *A. flavithermus* subsp. *flavithermus* DSM 2614**^T^**; 5, *A. eryuanensis* KCTC 13720**^T^**; 6, *A. tengchongensis* KCTC 13721**^T^**; 7, *A. pushchinoensis* DSM 12423**^T^**; 8, *A. thermarum* DSM 17141**^T^**; 9, *A. ayderensis* NCIMB 13972**^T^**; 10, *A. kamchatkensis* DSM 14988**^T^**; 11, *A. salavatliensis* DSM 22626**^T^**; 12, *A. kestanbolensis* NCIMB 13971**^T^**; 13, *A. contaminans* DSM 15866**^T^**; 14, *A. gonensis* NCIMB 13933**^T^**; 15, *A. voinovskiensis* DSM 17075**^T^**; 16, *A. kaynarcensis* LMG 25303^**T**^.

Data from *^a^, Dai et al. [10]; *^b^, Namsaraev et al. [13]; *^c^, Heinen et al. [3]; *^d^, Zhang et al. [12]; *^e^, Pikuta et al. [1]; *^f^, Poli et al. [8]; *^g^, Dulger et al. [5]; *^h^, Kevbrin et al. [9]; *^i^, Cihan et al. [11]; *^j^, De Clerck et al. [6]; *^k^, Belduz et al. [4]; *^l^, Yumoto et al. [7]; *^m^, Inan et al. [14]

S, spherical; E, ellipsoidal; O, oval; Cy, cylindrical; ST, subterminal; C, central; +, positive reaction; w, weakly positive reaction; -, negative reaction; ND, not determined.

**Figure 1:**
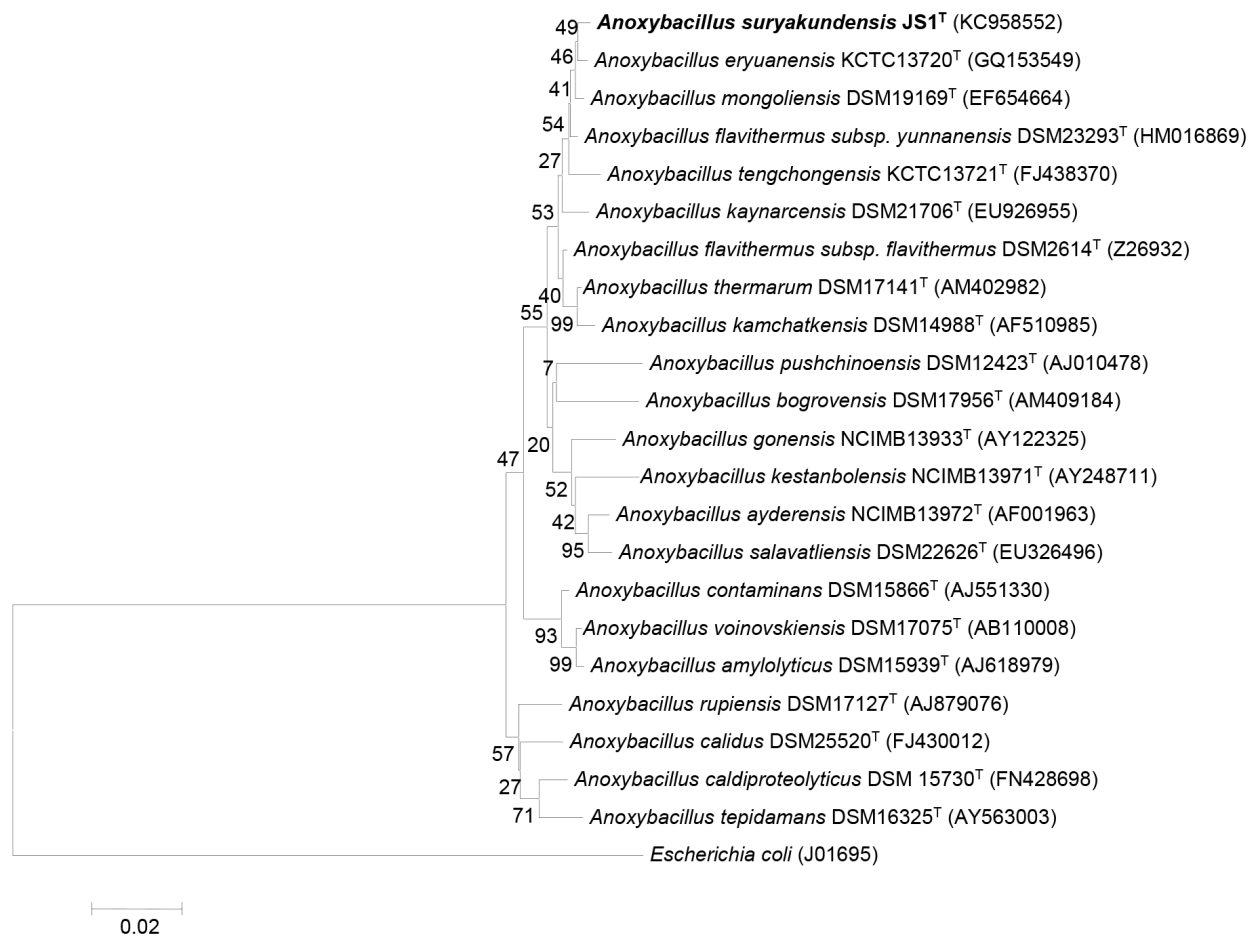
Neighbour-joining phylogenetic tree showing the position of *Anoxybacillus* *suryakundensis* strain JS1^T^ among the related taxa based on 16S rRNA secondary structure information. *E*. coli 16S rRNA sequence has been selected as reference template. Bootstrap values expressed as percentages of 1000 replications are given at branch points. Accession numbers are given in parentheses. Bar 2 substitutions per 100 nucleotide position.

16S rRNA sequence analysis revealed strain JS1^T^ showed 99.30% similarity with *A. flavithermus* subsp. *yunnanensis* DSM 23293^T^, 99.23% with *A. mongoliensis* DSM 19169^T^, 99.16% with *A. eryuanensis* KCTC 13720^T^, 98.74% with *A. flavithermus* subsp. *flavithermus* DSM 2614^T^, 98.54% with *A. tengchongensis* KCTC 13721^T^, 98.51% with *A. pushchinoensis* DSM 12423^T^, 97.91% with *A. thermarum* DSM 17141^T^, 97.82% with *A. kaynarcensis* LMG 25303^T^, 97.77% with *A. ayderensis* NCIMB 13972^T^, and *A. kamchatkensis* DSM 14988^T^, 97.63% with *A. salavatliensis* DSM 22626^T^, 97.55% with *A. kestanbolensis* NCIMB 13971^T^, 97.48% with *A. contaminans* DSM 15866^T^, 97.27% with *A. gonensis* NCIMB 13933^T^ and 97.17% with *A. voinovskiensis* DSM 17075^T^. In the phylogenetic tree, strain JS1^T^ clustered with *A. eryuanensis* KCTC 13720^T^ with 49% bootstrap confidence (Figure 1). The phylogenetic tree presented in this paper was similar in its topology to the tree generated by the maximum likelihood algorithm. Further, phylogenetic diversity was confirmed by comparing 16S rRNA secondary structures to find genus specific and species specific base substitutions or deletions (signature positions). By comparing 16S rRNA sequence of *A. suryakundnensis* strain JS1^T^, 15 species specific base substitutions (signature positions) were determined with those of the closely related strains. Maximum variability (8 out of 15 signatures) was confined within region 70-100 i.e. helix 6 (Table 2). 

**Table 2:** Species specific base substitutions in 16S rRNA secondary structure between *A. suryakundensis* strain JS1^T^ and phylogenetically closest members.

Signature	1	2	3	4	5	6	7	8
helix	6							
Position	71	72	77	78	90	92	93	96
*A. suryakundensis*	U	C	A	G	C	A	U	G
*A. eryuanensis*	C	G	A	A	U	G	A	C
*A. mongoliensis*	C	G	A	A	U	G	A	C
*A. flavithermus* subsp. *yunnanensis*	C	G	G	A	U	G	A	C
Signature	9	10	11	12	13	14	15	
helix	18		37		44		49	
Position	478	479	1011	1018	1134	1135	1453	
*A. suryakundensis*	U	G	A	U	A	C	C	
*A. eryuanensis*	U	A	A	U	A	C	U	
*A. mongoliensis*	C	U	G	U	A	C	C	
*A. flavithermus* subsp. *yunnanensis*	U	G	G	C	G	G	C	

The G+C content of strain JS1^T^ was 42.1 mol%, a value consistent with those of the genus *Anoxybacillus* (Table 1). 

In DNA-DNA hybridization study, using DNA of strain JS1^T^ as a labeled probe, the mean DNA-DNA re-association values were well below 70% for all type strains tested (Table S3). Higher re-association values were obtained with *A. flavithermus* subsp. *yunnanensis* DSM 23293^T^ (48.9±0.9%), A*. mongoliensis* DSM 19169^T^ (39±3.6%) and *A. eryuanensis* KCTC 13720^T^ (41.2±6.6%). Reciprocal probe were used in such cases that also indicated reliability of homology values. Therefore, given the recommended DNA–DNA relatedness cut-off point for species delineation of 70% [32], strain JS1^T^ should be regarded as a novel species of the genus *Anoxybacillus*. 

**Figure 2:**
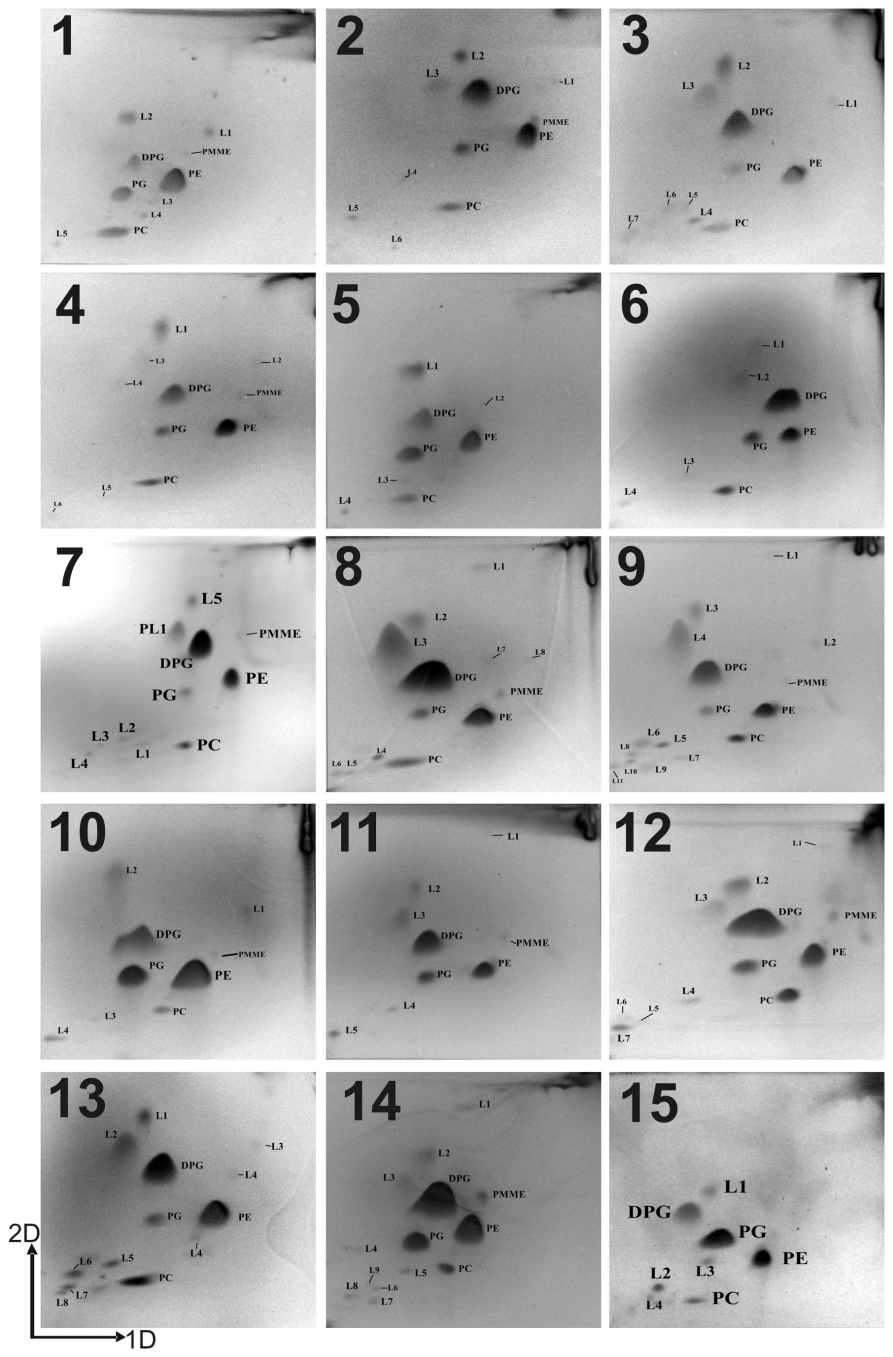
Polar lipid profile of strain JS1^T^ and its closely related species. Strains: 1, *A*. *suryakundensis* JS1^T^; 2, *A*. *flavithermus* subsp. *yunnanensis* DSM 23293^T^; 3, *A*. *mongoliensis* DSM 19169^T^; 4, *A*. *flavithermus* subsp. *flavithermus* DSM 2614^T^; 5, *A*. *eryuanensis* KCTC 13720^T^; 6, *A*. *tengchongensis* KCTC 13721^T^; 7, *A*. *thermarum* DSM 17141^T^; 8. *A*. *ayderensis* NCIMB 13972^T^; 9, *A*. *kamchatkensis* DSM 14988^T^; 10, *A*. *salavatliensis* DSM 22626^T^; 11, *A*. *kestanbolensis* NCIMB 13971^T^; 12, *A*. *contaminans* DSM 15866^T^; 13, *A*. *gonensis* NCIMB 13933^T^; 14, *A*. *voinovskiensis* DSM 17075^T^; 15, *A*. *kaynarcensis* LMG 25303^T^.

The major fatty acids of strain JS1^T^ were C_16:0iso,_ C_15:0iso_, C_17:1anteiso w9c_ and C_17:0iso_. Fatty acid compositions were almost identical to those of the closely related reference strains used in this study. However, strain JS1^T^ can be differentiated from other species by the absence of C_17:0anteiso_, C_17:1anteisoA_ and C_18:1w9c_ (Table 3). Polar lipids of strain JS1^T^ and the closely related reference strains comprised a phosphatidylgylycerol (PG), a diphosphatidylglycerol (DPG), a phosphatidylethnolamine (PE) and a phosphatidylcholine (PC) (Figure 2). Besides, strain JS1^T^ contained a phosphatidyl monomethylethanolamine (PMME) which was absent in *A. contaminans*, *A. gonensis*, *A. voinovskiensis*, and *A. tengchongensis*. In addition, several unidentified lipids were detected in the strain JS1^T^. Diaminopimelic acid was of meso type and glucose was sole sugar in cell wall in all strains that indicate genus uniformity. 

**Table 3:** Fatty acid profiles of *Anoxybacillus suryakundensis* strain JS1^T^ and the type strains of related *Anoxybacillus* species.

Peak Name	1	2	3	4	5	6	7*	8	9	10	11	12	13	14	15	16
12:0	tr	tr	tr	tr	tr	tr	6.9	tr	tr	tr	Tr	Tr	tr	tr	tr	1.75
12:0 aldehyde?	1.79	1.46	2.37	tr	tr	2.56	-	tr	4.16	-	Tr	2.65	tr	-	9.95	tr
14:0	tr	2.78	0.66	-	1.87	1.02	7.3	3.05	tr	1.02	1.10	2.02	1.13	1.09	1.24	1.09
14:0 3OH/16:1 iso I	1.79	1.46	2.37	tr	tr	2.56	-	tr	4.16	tr	tr	2.65	tr	1.26	9.95	tr
14:0 iso	tr	1.29	tr	tr	tr	1.87	-	tr	2.64	tr	tr	1.35	tr	1.95	5.25	tr
15:0 iso	17.96	34.59	42.16	54.80	56.64	59.36	38.70	48.44	55.44	57.06	60.92	57.07	56.48	53.79	37.94	52.34
15:0 anteiso	tr	4.16	tr	2.93	2.15	2.17	2.0	2.20	1.18	3.15	1.76	3.08	3.23	2.05	5.98	2.55
15:1 w5c	-	tr	-	tr	-	tr	-	0.58	tr	tr	tr	-	tr	tr	2.25	tr
16:0	tr	9.42	-	6.01	6.16	3.69	14.5	9.17	2.13	2.70	2.99	2.77	5.15	3.53	3.33	3.52
16:0 iso	56.47	9.47	24.09	tr	1.93	4.73	tr	1.40	10.84	1.71	1.29	2.33	3.29	8.29	13.22	1.92
16:1 w6c/16:1 w7c	1.20	9.44	2.00	5.96	2.04	1.77	-	5.73	3.14	3.41	3.10	6.45	1.88	2.13	2.40	2.32
16:1	-	-	-	-	-	-	2.6	-	-	-	-	-	-	-	-	-
16:1 2OH	1.03	-	tr	-	-	-	-	tr	-	-	-	-	-	-	-	-
17:0 iso	3.61	12.95	10.80	7.33	9.46	11.99	tr	9.67	8.33	11.32	11.05	5.01	14.05	13.84	6.39	11.62
17:0 anteiso	-	4.81	2.34	2.37	1.96	2.62	tr	2.98	1.02	3.30	1.43	1.62	3.54	2.91	3.30	3.32
17:1 iso w5c	1.61	3.47	5.52	5.56	2.91	3.33	-	4.89	5.50	6.59	8.72	8.67	4.40	3.35	3.50	5.59
17:1 anteiso A	-	1.95	-	1.58	tr	tr	-	1.87	tr	2.53	1.07	2.15	tr	tr	2.54	1.71
17:1 anteiso w9c	6.14	-	2.46	-	-	-	-	-	-	-	-	-	-	-	-	-
18:0	tr	tr	tr	1.13	-	tr	10.4	1.12	tr	tr	tr	-	tr	tr	tr	tr
18:1 2OH	tr	-	-	tr	tr	-	-	0.76	2.29	-	tr	-	tr	-	-	3.84
18:1 iso H	2.11	-	-	tr	-	-	-	-	-	-	-	-	-	-	-	-
18:1 w9c	-	tr	tr	4.04	3.76	tr	4.3	0.73	tr	1.15	tr	tr	tr	tr	tr	tr
18:2	-	-	-	-	-	-	2.2	-	-	-	-	-	-	-	-	-

Fatty acids were extracted from cells grown on tryptic soy agar plates at 55°C for 2 days. All data in the table are from the present study unless mentioned. Fatty acids amounting to less than 1.0% of the total fatty acids not mentioned in this table. Strains: 1, *A. suryakundensis*; 2, *A. flavithermus* subsp. *yunnanensis* DSM 23293^T^; 3, *A. mongoliensis* DSM 19169^T^; 4, *A. flavithermus* subsp. *flavithermus* DSM 2614^T^; 5, *A. eryuanensis* KCTC 13720^T^; 6, *A. tengchongensis* KCTC 13721^T^; 7, *A. pushchinoensis* DSM 12423^T^; 8, *A. thermarum* DSM 17141^T^; 9. *A. ayderensis* NCIMB 13972^T^; 10, *A. kamchatkensis* DSM 14988^T^; 11, *A. salavatliensis* DSM 22626^T^; 12, *A. kestanbolensis* NCIMB 13971^T^; 13, *A .contaminans* DSM 15866^T^; 14, *A. gonensis* NCIMB 13933^T^; 15, *A. voinovskiensis* DSM 17075^T^; 16, *A. kaynarcensis* LMG 25303^T^.

^*^ Data from Pikuta et al. [1].

tr, trace amount i.e. <1%; -, not detected.

Based on phylogentic, phenotypic and genotypic differences presented in this study by polyphasic approach, it can be concluded that strain JS1^T^ represents a novel species of genus *Anoxybacillus* for which the name *Anoxybacillus suryakundensis* is proposed.

### Description of *Anoxybacillus suryakundensis* sp. nov

*Anoxybacillus suryakundensis* (sur.ya.kund.en’sis. N.L. masc. adj. *suryakundensis* pertaining to Suryakund in Jharkhand, India, the geographical origin of isolation of the type strain). 

Cells are non motile, straight rod of length 1-4 µm and diameter 0.3-0.8 µm. Occurs single or in pair. Facultative anaerobe. Gram-positive. Endospores are spherical, located centrally or terminally and slightly swell sporangia. Aerobically grown colonies on TSA are pale yellow, rough surface, round with an uneven edge of diameter 1-2 mm. Moderately thermophilic and alkalitolerant. Grows at 40-60°C and pH 5.5-11.5 and can tolerate 3.5% NaCl. Optimum growth was observed at 55°C and pH 7.5. Catalase and oxidase positive. Positive for methyl red test and hydrolysis of gelatin, starch, esculin and DNA. Negative for reduction of nitrate, lysine decarboxylase, arginine dihydrolase, ornithine decarboxylase, phenylalanie deaminase, H_2_S production, indole, Voges Proskauer and hydrolysis of ONPG and urea. Acid is produced from oxidation of glucose, sucrose, fructose, trehalose, mannose, mannitol, lactose, maltose and starch. Negative for utilization of raffinose, galactose, xylose, rhamnose, malonate, citrate and arabinose. Cell wall contains glucose as sole sugar and DAP is of meso type. The major fatty acids are C_16:0iso_, C_15:0iso_, C_17:1anteisow9c_ and C_17:0iso_. Polar lipids include a phosphatidylgylycerol (PG), a diphosphatidylglycerol (DPG), a phosphatidylethnolamine (PE), a phosphatidylcholine (PC), a phosphatidyl monomethylethanolamine (PMME) and four unidentified lipids (L1-4). The G+C content of DNA is 42.1 mol%. *Anoxybacillus suryakundensis* strain JS1^T^, was isolated from a hot spring at Suryakund, Jharkhand, India.

## Supporting Information

Figure S1 **Dendrogram based on (**A**) ERIC-PCR and (**B**) REP-PCR.** The similarity between the species was calculated using the Pearson correlation coefficient for the range from 0.25 to 10 kb (optimization, 1%; position tolerance, 1%), and the species were grouped according to their similarities using UPGMA algorithm.
(TIF)

Figure S2 **Electron micrograph of strain JS1^T^.**
(TIF)

Figure S3 **Bright field image of strain JS1^T^ showing formation of endospore.** TS, terminal spore; CS, central spore; ES, exospores.
(TIF)

Table S1 **16S rRNA similarity and DNA-DNA homology values among strain JS1^T^ with other isolated strains.** DNA-DNA homology values are mean of two replicates. Standard deviation values are given in parentheses.
(DOCX)

Table S2 **Physiological and biochemical properties of isolated strains.**
(DOCX)

Table S3 **DNA-DNA homology values among strain JS1^T^ and closely related species of *Anoxybacillus*.** Reverse probe were used in cases when re-association values were intermediate i.e. near or above 40% Values are mean of two replicates. Standard deviation values are given in parentheses.
(DOCX)
