# Supplementary figures and images for: Anoxybacillus suryakundensis sp. nov, a Moderately Thermophilic, Alkalitolerant Bacterium Isolated from Hot Spring at Jharkhand, India
Source: PLoS One. 2013 Dec 20;8(12):e85493. doi: 10.1371/journal.pone.0085493 (PMC3869905; doi:10.1371/journal.pone.0085493)

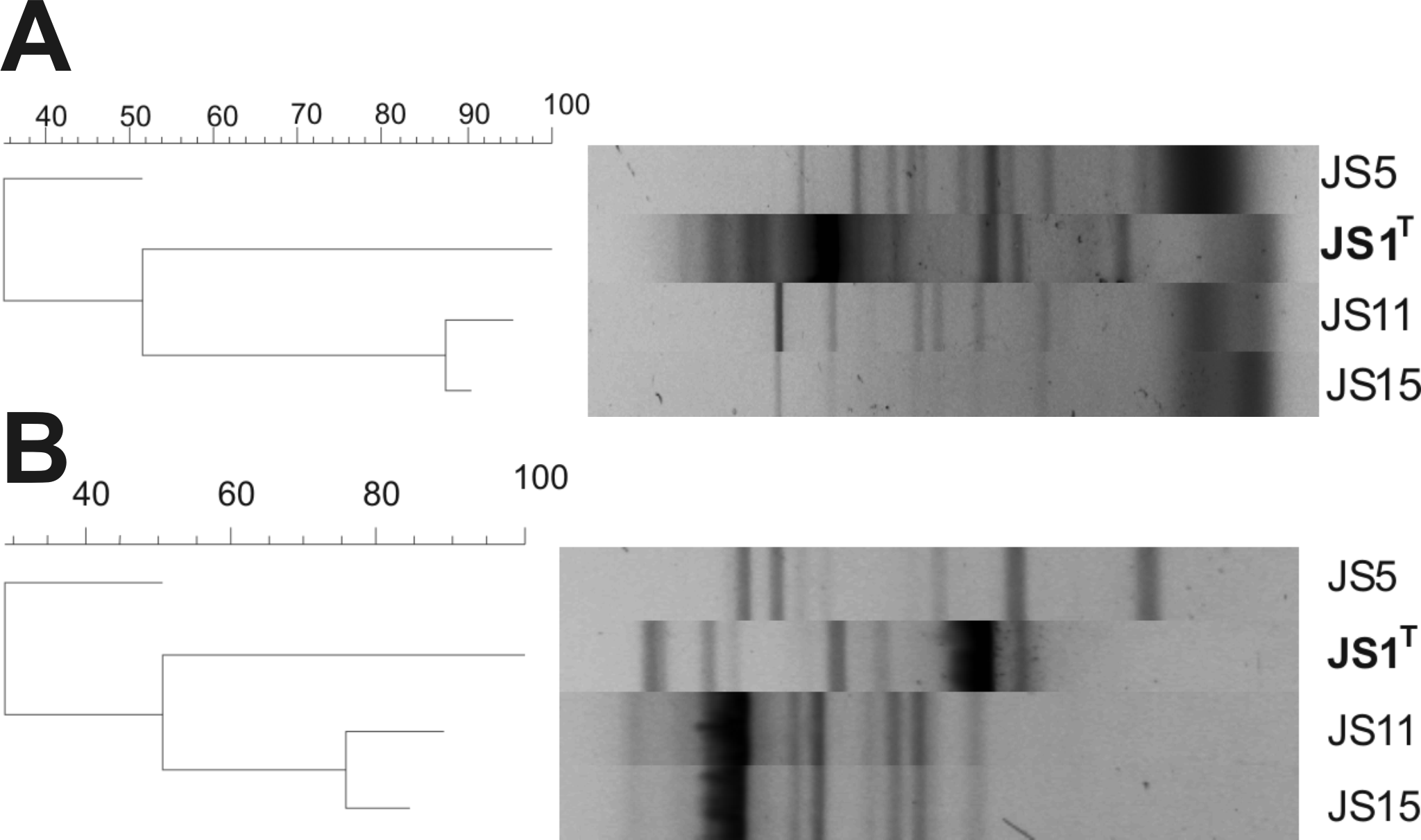

Supplement: Figure S1 — Dendrogram based on (A) ERIC-PCR and (B) REP-PCR. The similarity between the species was calculated using the Pearson correlation coefficient for the range from 0.25 to 10 kb (optimization, 1%; position tolerance, 1%), and the species were grouped according to their similarities using UPGMA algorithm. (TIF) [file pone.0085493.s001.tif]

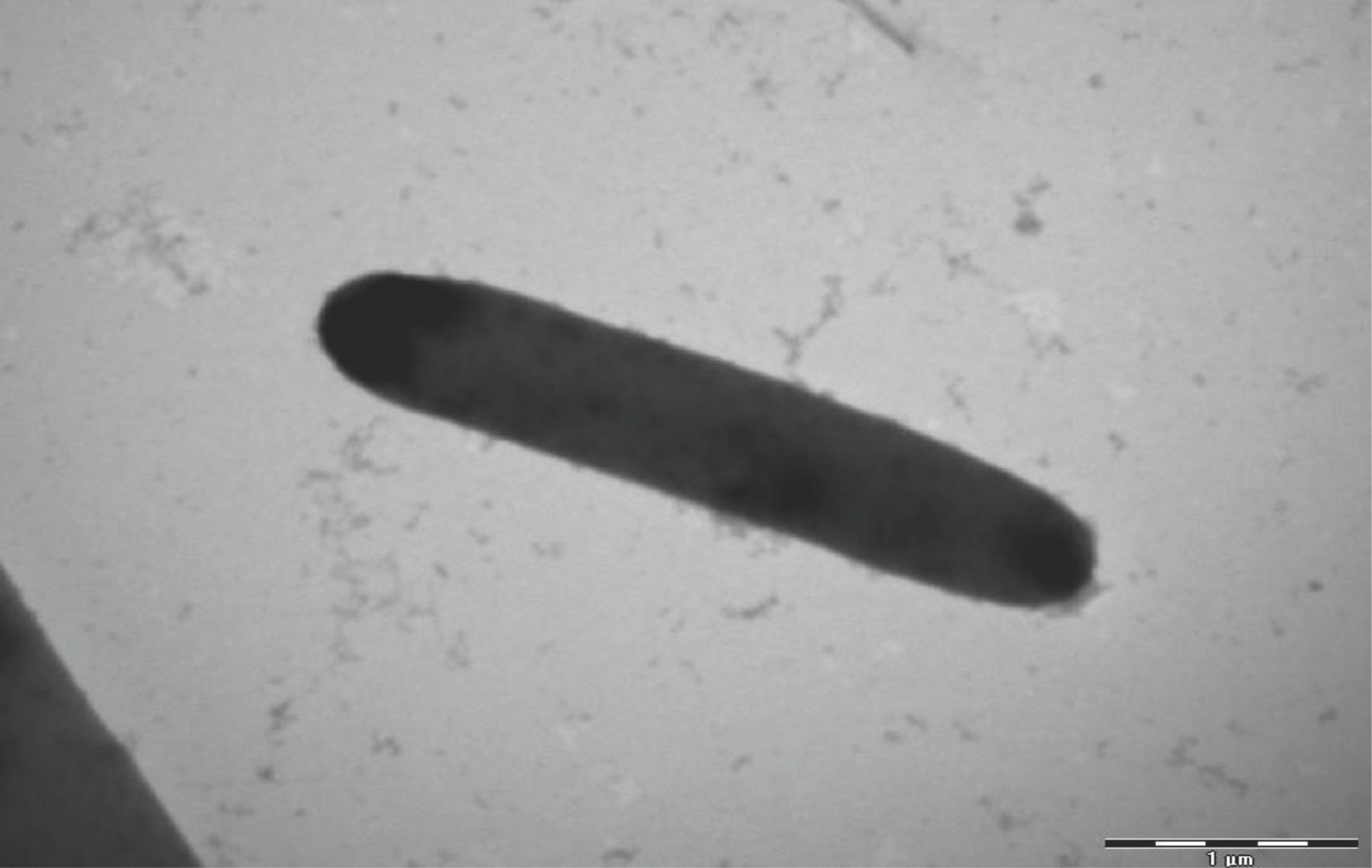

Supplement: Figure S2 — Electron micrograph of strain JS1T. (TIF) [file pone.0085493.s002.tif]

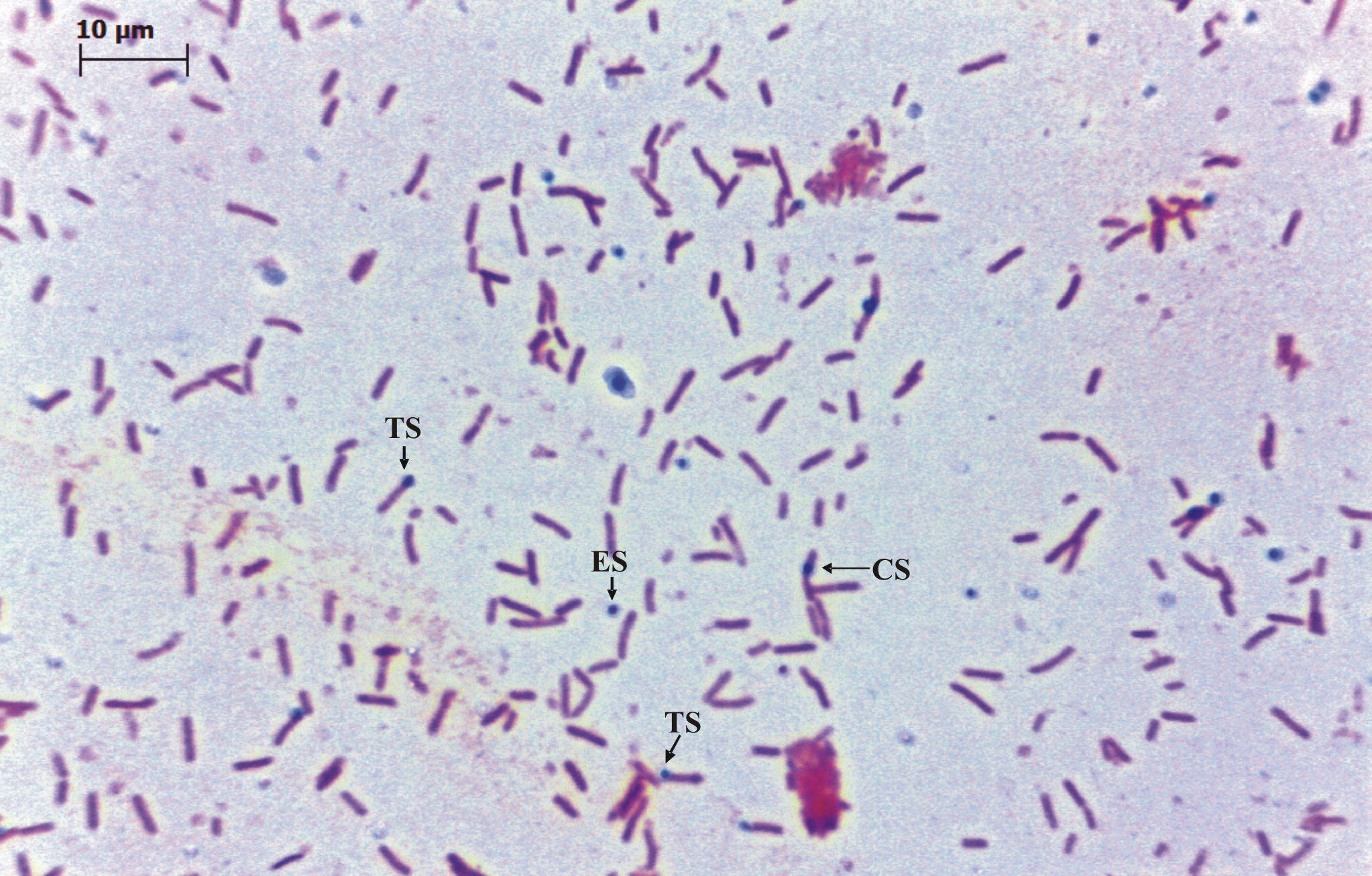

Supplement: Figure S3 — Bright field image of strain JS1T showing formation of endospore. TS, terminal spore; CS, central spore; ES, exospores. (TIF) [file pone.0085493.s003.tif]
