# Supplementary material for: Anoxybacillus suryakundensis sp. nov, a Moderately Thermophilic, Alkalitolerant Bacterium Isolated from Hot Spring at Jharkhand, India
Source: PLoS One. 2013 Dec 20;8(12):e85493. doi: 10.1371/journal.pone.0085493 (PMC3869905; doi:10.1371/journal.pone.0085493)
Supplement: Table S1 — 16S rRNA similarity and DNA-DNA homology values among strain JS1T with other isolated strains. DNA-DNA homology values are mean of two replicates. Standard deviation values are given in parentheses. (DOCX) [file pone.0085493.s004.docx]

**Table S1.** 16S rRNA similarity and DNA-DNA homology values among strain JS1^T^ with other isolated strains. DNA-DNA homology values are mean of two replicates. Standard deviation values are given in parentheses.

| Strains | 16S rRNA similarity | | | | DNA-DNA hybridization values (%) | | | |
| --- | --- | --- | --- | --- | --- | --- | --- | --- |
|  | JS1 | JS5 | JS11 | JS15 | JS1 | JS5 | JS11 | JS15 |
| JS1 | 100 | 99.16 | 97.13 | 97.98 | 100 | 74.8 (0.1) | 98.2 (1) | 73.4 (1.4) |
