# Supplementary material for: Anoxybacillus suryakundensis sp. nov, a Moderately Thermophilic, Alkalitolerant Bacterium Isolated from Hot Spring at Jharkhand, India
Source: PLoS One. 2013 Dec 20;8(12):e85493. doi: 10.1371/journal.pone.0085493 (PMC3869905; doi:10.1371/journal.pone.0085493)
Supplement: Table S2 — Physiological and biochemical properties of isolated strains. (DOCX) [file pone.0085493.s005.docx]

**Table S2.** Physiological and biochemical properties of isolated strains.

| Features | JS1^T^ | JS5 | JS11 | JS15 |
| --- | --- | --- | --- | --- |
| Motility | - | - | - | - |
| Biochemical tests |  |  |  |  |
| Catalase | + | + | + | + |
| Oxidase | + | + | + | + |
| Nitrate reduction | **-** | **-** | **-** | **-** |
| Methyl red | + | + | + | + |
| Growth characteristics |  |  |  |  |
| Temperature range (°C) | 40-60 | 40-60 | 40-60 | 40-60 |
| Temperature optima (°C) | 55 | 55 | 55 | 55 |
| pH range | 5.5-11.5 | 5.5-11 | 5.5-10 | 5.5-10 |
| pH optima | 7.6 | 7.6 | 7.6 | 7.6 |
| NaCl tolerance (%) | 3.5 | 4 | 3 | 3 |
| Ethanol tolerance (%) | 2 | 2 | 1 | 1 |
| Biochemical tests |  |  |  |  |
| Catalase, oxidase, methyl red | + | + | + | + |
| Indole, Voges Proskauer, H_2_S production, arginine dihydrolase, ornithine decarboxylase, lysine decarboxylase, phenylalanine deaminase and urease | - | - | - | - |
| Hydrolysis of |  |  |  |  |
| Gelatin, Esculin, DNA | + | + | + | + |
| Starch | + | + | - | - |
| Acid production from D-glucose | + | + | + | + |
| Utilization of |  |  |  |  |
| D-glucose, D-fructose, D-mannose, D-mannitol, Esculin, D-maltose, D-sucrose, D-trehalose, glycogen | + | + | + | + |
| L-rhamnose, D-raffinose, galactose | - | - | - | - |
| Glycerol | w | + | + | + |
| L-arabinose | - | + | w | - |
| D-xylose | - | + | - | + |
| Arbutin | - | + | - | - |
| Salicin | w | + | + | + |
| D-cellobiose | w | + | + | + |
| Lactose | w | w | + | - |
| Starch | + | + | - | - |
| D-turanose | w | w | - | w |
| Potassium 5-ketogluconate | w | w | + | w |
| G+C content (mol%) | 42.1 | ND | ND | ND |

+, positive reaction; w, weakly positive reaction; -, negative reaction; ND, not determined
