# Supplementary material for: Anoxybacillus suryakundensis sp. nov, a Moderately Thermophilic, Alkalitolerant Bacterium Isolated from Hot Spring at Jharkhand, India
Source: PLoS One. 2013 Dec 20;8(12):e85493. doi: 10.1371/journal.pone.0085493 (PMC3869905; doi:10.1371/journal.pone.0085493)
Supplement: Table S3 — DNA-DNA homology values among strain JS1T and closely related species of Anoxybacillus. Reverse probe were used in cases when re-association values were intermediate i.e. near or above 40% Values are mean of two replicates. Standard deviation values are given in parentheses. (DOCX) [file pone.0085493.s006.docx]

**Table S3.** DNA-DNA homology values among strain JS1^T^ and closely related species of *Anoxybacillus*. Reverse probe were used in cases when re-association values were intermediate i.e. near or above 40% Values are mean of two replicates. Standard deviation values are given in parentheses.

| Probe | DNA-DNA homology with | | | | | | | | | | | | | | |  |
| --- | --- | --- | --- | --- | --- | --- | --- | --- | --- | --- | --- | --- | --- | --- | --- | --- |
|  | 1 | 2 | 3 | 4 | 5 | 6 | 7 | 8 | 9 | 10 | 11 | 12 | 13 | 14 | 15 | 16 |
| 1 | 100 | 48.9 (0.9) | 39 (3.6) | 41.2 (6.6) | 26.2 (0.1) | 13.5 (1.2) | 19.5 (5.0) | 8.1 (3.9) | 27.3 (2.6) | 28.4 (1.76) | 3.5 (0.1) | 5 (4.2) | 18.8 (6.2) | 17.8 (4.6) | 19.9 (2.2) | 17.9 (1.4) |
| 2 | 55.8 (0.8) | 100 |  |  |  |  |  |  |  |  |  |  |  |  |  |  |
| 3 | 23.7 (1) |  | 100 |  |  |  |  |  |  |  |  |  |  |  |  |  |
| 4 | 43.9 (1.9) |  |  | 100 |  |  |  |  |  |  |  |  |  |  |  |  |

Strains: 1, *A. suryakundensis* strain JS1^T^; 2, *A. flavithermus* subsp. *yunnanensis* DSM 23293^T^; 3, *A. mongoliensis* DSM 19169^T^; 4, *A. eryuanensis* KCTC 13720^T^; 5, *A. flavithermus* subsp. *flavithermus* DSM 2614^T^; 6, *A. tengchongensis* KCTC 13721^T^; 7, *A. pushchinoensis* DSM 12423^T^; 8, *A. thermarum* DSM 17141^T^; 9, *A. ayderensis* NCIMB 13972^T^; 10, *A. kamchatkensis* DSM 14988^T^; 11, *A. salavatliensis* DSM 22626^T^; 12, *A. kestanbolensis* NCIMB 13971^T^; 13, *A. contaminans* DSM 15866^T^; 14, *A. gonensis* NCIMB 13933^T^; 15, *A. voinovskiensis* DSM 17075^T^; 16, *A. kaynarcensis* LMG 25303^T^.
